# Neurological mechanism and treatment effects prediction of acupuncture on migraine without aura: Study protocol for a randomized controlled trial

**DOI:** 10.3389/fneur.2022.981752

**Published:** 2022-09-08

**Authors:** Jiahui Hong, Jingqing Sun, Liping Zhang, Zhongjian Tan, Ying Chen, Qiuyi Chen, Yupu Zhu, Yuhan Liu, Liying Zhu, Lin Zeng, Yazhuo Kong, Bin Li, Lu Liu

**Affiliations:** ^1^School of Clinical Medicine, Beijing University of Chinese Medicine, Beijing, China; ^2^Department of Acupuncture and Moxibustion, Beijing Hospital of Traditional Chinese Medicine, Capital Medical University, Beijing Key Laboratory of Acupuncture Neuromodulation, Beijing, China; ^3^Department of Radiology, Dong Zhimen Hospital Beijing University of Chinese Medicine, Beijing, China; ^4^Department of Psychology, University of Chinese Academy of Sciences, Beijing, China; ^5^Peking University Third Hospital, Research Centre of Clinical Epidemiology, Beijing, China

**Keywords:** acupuncture, clinical trial, migraine without aura, multimodal magnetic resonance imaging, neurological mechanism, protocol, treatment effects prediction

## Abstract

**Introduction:**

Acupuncture is an effective treatment in migraine without aura (MWoA), but the neurological mechanism has not been investigated using multimodal magnetic resonance imaging (MRI). This trial will combine functional MRI, structural MRI, and diffusion tensor imaging to explore the potential neural mechanism of acupuncture on MWoA, and will use machine learning approach to predict acupuncture treatment effects.

**Methods:**

In this multimodal neuroimaging randomized controlled trial, a total of 60 MWoA participants will be randomly allocated to two groups: the real acupuncture treatment group and the sham acupuncture control group. This trial will include a 4-week baseline phase, a 4-week treatment phase, and a 12-week follow-up phase. Participants will undergo 12 acupuncture or sham acupuncture sessions during the treatment phase. The Headache Diary, Migraine-Specific Quality of Life Questionnaire, Headache Impact Test, Beck Depression Inventory-II, and Beck Anxiety Inventory will be utilized to evaluate the clinical efficacy. Multimodal MRI scans will be employed to investigate the mechanism of acupuncture at baseline, at the end of treatment, and after follow-up. Multimodal MRI data will be used to predict acupuncture treatment effects using machine learning technology.

**Discussion:**

This study hypothesized that acupuncture therapy may treat MWoA by restoring the neuropathological alterations in brain activity. Our finding should provide valuable scientific proof for the effects of acupuncture and demonstrate the usefulness of acupuncture in the treatment of MWoA. Moreover, acupuncture response prediction might decrease healthcare expenses and time lags for patients.

**Trial registration number:**

[ChiCTR2100044251].

## Introduction

Migraine is a debilitating condition characterized by episodic or chronic headaches and physiological and emotional stressors (1, 2) that represents an important healthcare and social problem (3, 4). Migraine is a recurrent brain function disorder that affects approximately one billion individuals worldwide, from all geographies, ethnicities, and socio-economic backgrounds (5, 6). The 1-year prevalence of migraine is reported to be 15% globally, being the greatest in Southeast Asia (25–35%), and the lowest in China (9%) (7, 8).

The current armamentarium of treatment options includes acute, preventive, and non-pharmacological therapies. Acupuncture is traditionally used as a non-pharmacological therapy to treat various symptoms and diseases. Acupuncture is widely used for its analgesic benefits due to its effectiveness and safety (9). There is moderate evidence for acupuncture being at least non-inferior to conventional drug therapy (e.g., flunarizine, metoprolol) for episodic migraine prevention (10, 11). A recent study compared acupuncture with the most appropriate medication for each patient, which came out a conclusion that for migraine prophylaxis, acupuncture is just as successful as the suitable pharmaceutical therapy (12). However, acupuncture's clinical effects have been challenged on the basis that some of its effects may relate to psychological or ‘placebo’ mechanisms. Fortunately, plenty clinical evidence suggests that real acupuncture is superior to sham acupuncture in migraine, reflected by a higher total effective rate and decreased recurrence rate (13). Two previous meta-analyses recommended acupuncture for the prevention of episodic migraine (10, 14). Acupuncture is also recommended by guidelines in many countries for the management and therapy of migraine (15–21).

Due to the wide applications, growing attention has been paid to discovering rational explanations for the complicated neurophysiological mechanisms of acupuncture. Acupuncture has been proven in several studies to change a number of neurophysiological factors related to analgesia, which are considered to reduce or modulate pain perception and physiological processes (10). In addition, acupuncture has been proved to participate in the development of peripheral and central sensitization through modulation of the release of related mediators and serotonin system activation (22–24). Neuroimaging studies also have indicated that acupuncture could alter the abnormal functional activity and connectivity of the descending pain modulatory system, default mode network, and several related subcortical areas (25–27). Nevertheless, the complicated neurophysiological mechanisms of acupuncture for migraine prevention should be investigated independently (28). Moreover, no research have been conducted to investigate the mechanism underlying acupuncture's efficacy on migraine without aura (MWoA) using multimodal magnetic resonance imaging (MRI), which combines functional MRI (fMRI), structural MRI (sMRI), and diffusion tensor imaging (DTI).

Although acupuncture is used to treat migraine across many countries (9, 10, 29–34), approximately 50% of the migraineurs do not obtain significant relief following acupuncture (33, 34). The capacity to predict the effects of acupuncture could save the non-responders from having to suffer extended periods of ineffective therapy. Our group's most recent study used baseline brain gray matter volume as a predictor of acupuncture outcomes in migraine treatment (35). The machine learning classification approach is increasingly being implemented to identify patient subgroups or to predict responders and non-responders with certain therapies (36–38), giving a new paradigm for personalized therapeutic strategies.

Hence, we designed this multimodal brain imaging trial with three aims: (1) to re-evaluate the efficacy of acupuncture treatment for MWoA by comparing real acupuncture with sham acupuncture, (2) to investigate the promising neural mechanism of acupuncture for MWoA by integrating multimodal MRI, and (3) to apply machine learning approach to predict the effects of acupuncture on MWoA.

## Methods

### Study design

**Figure 1:**
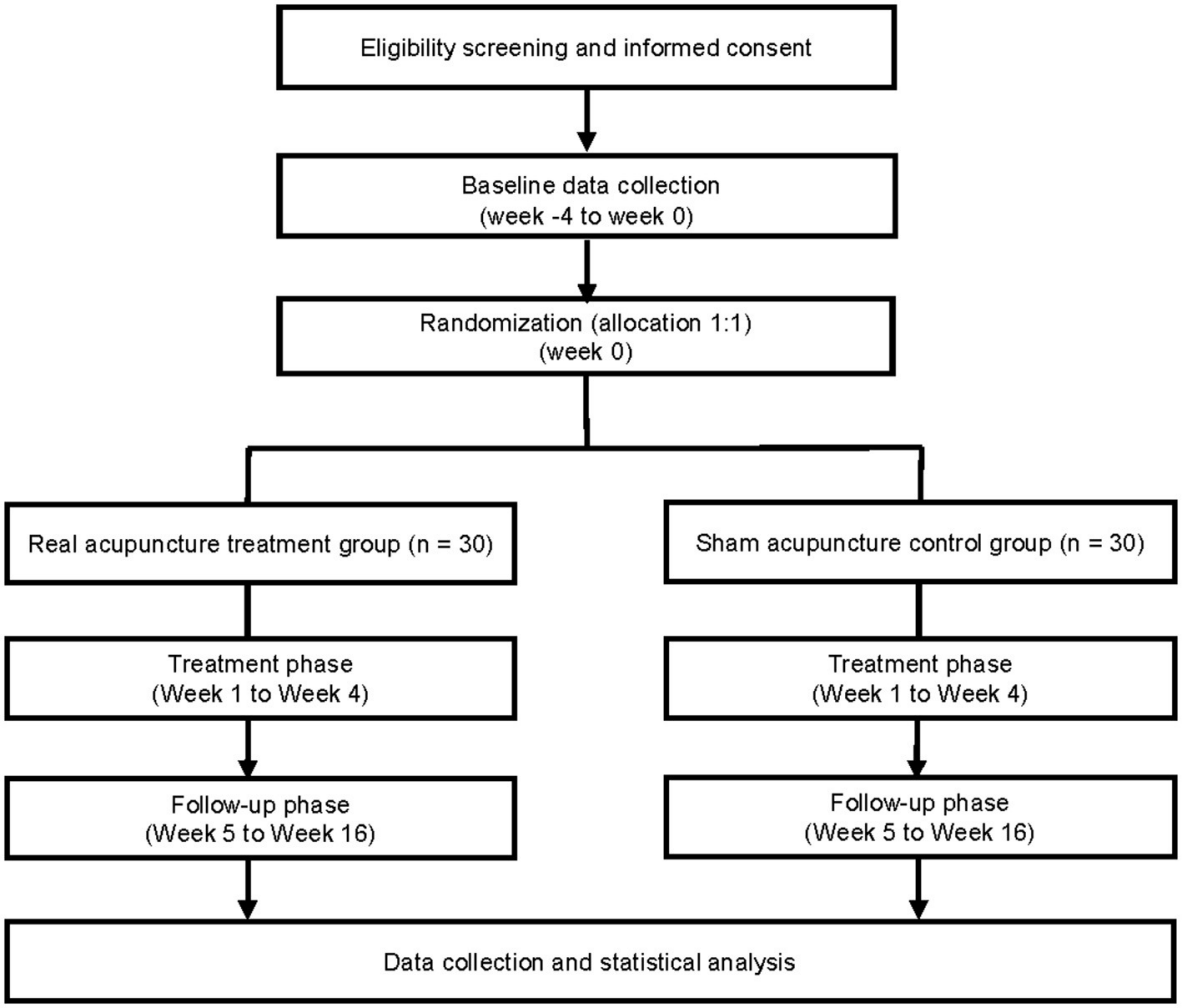
Study flowchart.

This trial was designed to evaluate the treatment effect and neurological mechanism of acupuncture in MWoA. Our study will be conducted as a 1:1 randomized, single-blinded, and sham-controlled study. Sixty participants diagnosed with MWoA will be considered eligible. They will be randomly assigned to two groups with 30 participants each. The real acupuncture treatment group will receive real acupuncture, whereas the sham acupuncture control group will undergo sham acupuncture. Pain, neuropsychological questionnaires, and multimodal MRI evaluation will be performed at baseline, after the treatment, and during the follow-up phase. This study will consist of three phases: (i) a 4-week screening phase (week−4 to week 0); (ii) a 4-week treatment phase (weeks 1–4), and (iii) a 12-week follow-up phase (weeks 5–16). At baseline, participants will be assessed *via* headache diaries, the Migraine-Specific Quality of Life Questionnaire (MSQ), Headache Impact Test (HIT-6), Beck Depression Inventory-II (BDI-II), Beck Anxiety Inventory (BAI), and multimodal MRI scans. The enrollment schedules, interventions, and assessments are summarized in Table 1, and a study flowchart is presented in Figure 1.

**Table 1:** The enrolment schedules, interventions, and assessments.

	**Study period**								
	**Enrolment**	**Allocation**	**Treatment phase**				**Follow-up phase**		
**Timepoint**	**–4 week**	**0 week**	**1 week**	**2 week**	**3 week**	**4 week**	**8 week**	**12 week**	**16 week**
**Enrollment**									
Eligibility screen	×								
Informed consent	×								
Allocation		×							
**Interventions**									
Real acupuncture treatment group			×	×	×	×			
Sham acupuncture control group			×	×	×	×			
**Assessments**									
Headache diaries	×	×	×	×	×	×	×	×	×
Number of monthly headache onset		×				×	×	×	×
Monthly headache days		×				×	×	×	×
Mean VAS score		×				×	×	×	×
Headache duration (h)		×				×	×	×	×
Percentage of participants taking acute pain medication		×				×	×	×	×
Treatment responder rate		×				×	×	×	×
MSQ		×				×			×
HIT-6		×				×			×
BDI-II		×				×	×	×	×
BAI		×				×	×	×	×
MRI scan		×				×			×
**Participants safety**									
Adverse events			×	×	×	×	×	×	×

VAS, visual analog scale; MSQ, Migraine-Specific Quality of Life Questionnaire; HIT-6, Headache Impact Test; BDI-II, Beck Depression Inventory-II; BAI, Beck Anxiety Inventory.

This trial will be reported following the Standard Protocol Items: Recommendations for Intervention Trials (SPIRIT) guidelines (39) (Supplementary material) and will follow the Consolidated Standards of Reporting Trials (CONSORT) (40) and the Standards for Reporting Interventions in Clinical Trials of Acupuncture principles (STRICTA) (41).

### Recruitment

The researchers will recruit trial participants with MWoA from outpatient clinics at the Acupuncture and Moxibustion Department of the Beijing Hospital of Traditional Chinese Medicine, Capital Medical University. A certified neurologist (LL) will perform the diagnosis of MWoA and exclude other types of headaches or diseases based on syndromes and accessory examinations. To promote recruitment, advertisements will be posted in the hospital. The recruitment advertisement for the study will also be posted in the public account of WeChat of our hospital. For comparison with the MRI data analysis, 30 age- and sex-matched healthy controls will be recruited to receive a multimodal MRI scan. Researchers who are not involved in the treatment process will conduct the entire recruitment procedure.

Participants will be explained the purpose and nature of the study protocol. Written informed consent will be obtained from all the participants. Each participant will be informed of the random allocation of real acupuncture or sham acupuncture, and the potential benefits and risks. They will voluntarily sign an informed consent form prior to enrollment. Participants will be able to withdraw from this research at any time, without penalty or loss of benefits, and without having to give a reason. Meanwhile, we will endeavor to record the reason for withdrawal.

### Participants

#### Inclusion criteria for patients with MWoA

The inclusion criteria will be as follows: (1) patients that fulfill the diagnostic criteria of the International Classification of Headache Disorders, 3rd edition for MWoA (33); (2) a migraine history of at least 1 year before study entry; (3) having experienced at least two migraine attacks in the preceding 4 weeks; (4) aged between 18 and 65 years, right-handed; (5) not having received treatments like acupuncture or pharmacological medicine (e.g., paracetamol, triptans, amitriptyline, beta-blockers, topiramate, valproic acid, or anti-GCRP monoclonal antibodies) in the 3 months before enrollment; (6) able to complete a headache diary.

#### Exclusion criteria for patients with MWoA

We will exclude patients with (1) chronic migraine, tension-type headache, cluster headache, or other primary headaches; (2) secondary headache caused by otorhinolaryngological diseases or intracranial pathological changes; (3) relatively severe systemic diseases (e.g., angiocardiopathy, cerebrovascular disease, hepatopathy, nephropathy, acute infectious disease, hematopathy, endocrinopathy, allergy, or methysis); (4) pregnancy, lactation, or insufficient contraception; (5) psychiatric or neurological disorders (e.g., major depressive disorder, schizophrenia, bipolar disorder, traumatic brain injury, stroke, Parkinson's disease); or (6) MRI contraindications (e.g., claustrophobia, cardiac pacemaker, or other metallic objects implanted in the body).

#### Inclusion criteria for healthy controls

The group of healthy controls will consist of participants (1) aged 18 to 65 years, right-handed; (2) with no personal or familial history of migraine, and (3) who agree to participate in this study voluntarily and to sign an informed consent form.

#### Exclusion criteria for healthy controls

(1) Relatively severe systemic diseases (e.g., angiocardiopathy, cerebrovascular disease, hepatopathy, nephropathy, acute infectious disease, hematopathy, endocrinopathy, allergy, or methysis) (2) pregnancy, lactation, or insufficient contraception (3) psychiatric or neurological disorders (e.g., major depressive disorder, schizophrenia, bipolar disorder, traumatic brain injury, stroke, Parkinson's disease), or (4) MRI contraindications (e.g., claustrophobia, cardiac pacemaker, or other metallic objects implanted in the body).

### Randomization and allocation concealment

Block randomization will be employed to achieve a balance between the two groups, with a block size of four. Predetermined, sequentially numbered, opaque, and sealed envelopes will be produced by a third-party professional statistician (LZe) from the Epidemiology Research Center of Peking University Third Hospital using the PROC PLAN of Statistical Analysis System (SAS) 9.4 software (SAS Institute Inc., Cary, NC, USA) to avoid subjective bias from the researchers. Each sealed envelope will be numbered sequentially and kept secure until the trial is finished.

An independent assistant will separate and unseal each envelope from the strain in the sequence corresponding to the participant's screening order and will then assign eligible participants to the real acupuncture treatment group or the sham acupuncture control group. The assistant will not be involved in the baseline or outcome assessments. The treatment allocation results will be forwarded to the acupuncturist by this assistant. With the exception of the acupuncturist and the assistant who unsealed the envelopes, neither the participants nor the other researchers will be aware of group allocation.

### Blinding

During the trial, participants will not be told which treatment they will receive: real acupuncture or sham acupuncture. To eliminate meaningful communication between the two groups during treatment, participants will receive acupuncture therapy in consulting rooms with compartments. Therefore, participants will be blinded to the treatment allocation. The acupuncturist will not be blinded to the group allocation; they will be unable to disclose the type of treatment.

Furthermore, outcome assessors, MRI scanners, and statisticians will be blinded to the treatment allocation of the participants throughout the trial. To avoid outcome assessors and statisticians from identifying that how participants are allocated, the labels of the real acupuncture treatment group and the sham acupuncture control group will be substituted with non-sensical letter combinations. Unblinding will be performed only after the trial finishes except if the participant has an adverse event suspected to be related to the acupuncture treatment (42).

The credibility of the blinding will be assessed at the end of the treatment phase (week 4). An investigator who is not engaged in the participant allocation or the therapy administration will examine the credibility of the participants' blinding.

### Intervention

#### Real acupuncture treatment group

**Figure 2:**
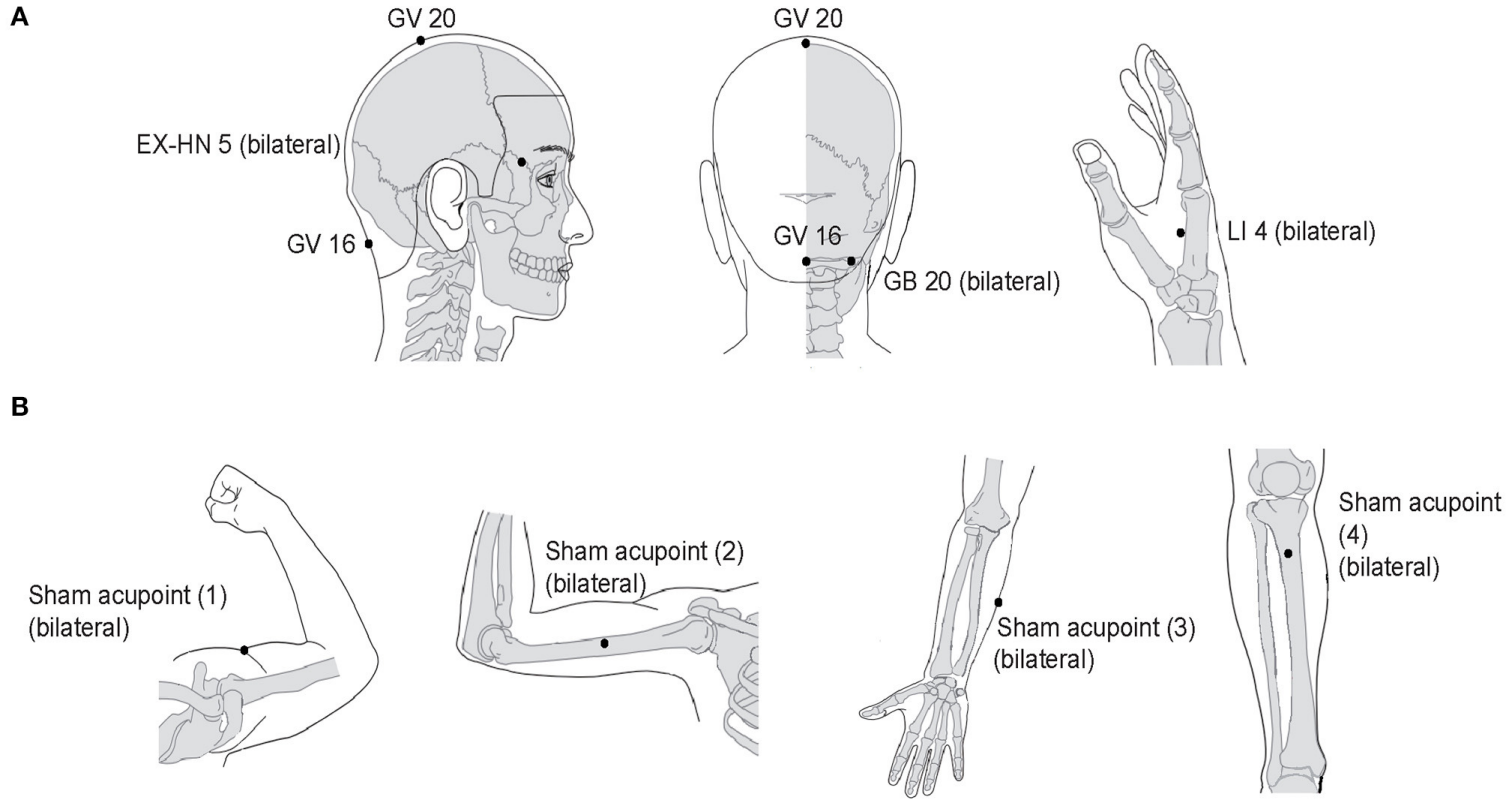
Location of real acupoints and sham acupoints. **(A)** Real acupoints. **(B)** Sham acupoints. EX-HN, Extrachannel points; GB, Gallbladder Meridian; GV, Governor Vessel; LI, Large Intestine Meridian.

Participants in this group will obtain manual acupuncture at eight real acupoints (bilateral Fengchi [GB20], Fengfu [GV16], Baihui [GV20], bilateral Taiyang [EX-HN5], and bilateral Hegu [LI4]) (Figure 2) (Supplementary material) with disposable single-use sterile needles (0.25 × 25 mm, Ande Medical Instrument Co., Ltd., China). According to records in ancient and modern books, real acupoints have been proven effective and crucial, the suggestion of consensus meetings with clinical experts, and the results of our previous research on acupuncture treatment for MWoA (34). The positioning of acupoints will be determined according to the standards issued by the World Health Organization in 2010 (43). Needles will be inserted into acupoints at a depth of 10–15 mm following skin disinfecting with alcohol; the acupuncturist will twist the needles by 90–180°, lifting and thrusting needles with an amplitude of 3–5 mm to generate a deqi sensation. After achieving deqi sensation, the needles will be held at the acupoints for 30 min. The foregoing procedures will be manipulated for 10–15 s every 10 min to sustain the deqi sensation. Participants will obtain a total of 12 sessions of acupuncture treatment for 4 weeks, with three sessions per week.

#### Sham acupuncture control group

This group will obtain superficial skin penetration at eight sham acupoints. To minimize physiological effects, these sham acupoints do not correspond to any known meridian and are not conventional acupoints according to previous studies (44, 45). The sham acupoint locations are shown in Figure 2 (Supplementary material). Superficial skin penetration at sham acupoints, without needle manipulation for deqi sensation, is a common sham acupuncture control applied in many randomized controlled trials (46). Participants in sham group will receive an acupuncture procedure similar to that received by the participants in the real acupuncture treatment group. Additionally, the number and size of sterile steel needles for each treatment session will be the same as in the real acupuncture treatment group.

The same acupuncturist will perform therapy to minimize the bias caused by the different personal practices of the acupuncturists. The acupuncturist is a practicing physician licensed by the Ministry of Health of the People's Republic of China. The acupuncturist has more than five years of clinical experience. Before the commencement of the study, the acupuncturist will undergo specific training on the trial's purpose and standardized procedures, therapeutic interventions, and quality management.

The participants' weekly acupuncture times and compliance will be recorded in detail in case report forms (CRFs). Moreover, the number of acupuncture treatments received by each participant will be counted to monitor patient adherence.

#### Concomitant medication

Patients will be instructed to avoid taking medications as much as possible during the trial. According to principles of medical ethics, if the participants' migraine symptoms do not improve considerably, and they are unable to endure migraine attack, ibuprofen may be temporarily prescribed. Ibuprofen (300 mg per capsule with sustained release) will be recommended to all participants as their first option for acute medication. Except for ibuprofen, no drugs will be taken in this study. Researchers will record the name, dose, and time of the drugs taken in headache diaries and CRFs.

### Multimodal data acquisition

All participants will receive an MRI scan in the Department of Radiology, Dong Zhimen Hospital Beijing University of Chinese Medicine, after providing their informed consent. MRI data will be obtained with a 3.0 Tesla superconductor (Siemens Magnetom Verio, Erlangen, Germany). Participants in both the real acupuncture treatment group and sham acupuncture control group will be scanned before randomization, at the end of treatment, and after the follow-up phase. In contrast, healthy controls will be scanned after recruitment. Given the possibility of variations associated with the menstrual cycle, female participants will be assessed outside the premenstrual or menstrual phases. All participants will undergo the MRI during the headache-free interval (not within 48 h after the last migraine attack). During scanning, the participants will be required to lie on their backs in the scanner. A headrest will be used to hold the head to prevent head movements from affecting the image, and a 16-channel head coil will be fixed on both sides of the headrest. They will be instructed to stay awake, relax, and not think of anything during the entire brain scan, with their ears plugged to attenuate the gradient noise. During fMRI scanning, participants will be instructed to view a centrally placed fixation cross (+), and during T1 and DTI scanning, they will be allowed to close their eyes and relax. Furthermore, the MRI scan will be conducted by the same technician (YZhu) using the same machine, and a professional staff member (ZT) will review the images for quality and procedure compliance after each scan. Both of them are highly qualified radiologists skilled in MRI.

First, the scanning procedure produces a T1-weighted structural image (repetition time (TR)/echo time (TE) = 2,000/3.51 ms, slice thickness = 1.00 mm, slice number =192, field of view (FOV) = 256 × 256 mm, flip angle = 8°, matrix size = 256 × 256) for standard space co-registration. Second, a T2^*^-weighted gradient-echo (GE) blood oxygenation level-dependent (BOLD) sequence will be recorded (TR/TE = 2,420/30 ms, slice thickness = 3.0 mm, slice number = 44, FOV = 192 × 192 mm, flip angle = 90°, matrix size = 64 × 64) to obtain fMRI data. Third, a DTI sequence (TR/TE, 12,700/91 ms; slice thickness = 2.0 mm, slice number = 64, FOV = 192 × 192 mm; matrix size = 96 × 96) will be used to collect connectivity information (Supplementary material). Scanning will be terminated if the participant complains of discomfort. A professional staff member (ZT) will check the images qualitatively to detect for any brain lesions or structural abnormalities.

### Outcome measures

#### Primary outcome measure

The primary outcome is the change in the number of monthly headache onsets from week 0 to week 4.

#### Secondary outcome measures

The secondary outcome measures are as follows: (1) change in the number of monthly headache onsets from week 0 to week 16; (2) change in monthly headache days (MMDs); (3) change in visual analog scale (VAS) score of headache attack intensity; (4) change in headache duration (h); (5) percentage of participants taking acute pain medication; (6) treatment responder rate (the proportion of participants with ≥50% reduction in the number of monthly headache onsets); (7) change in MSQ (8, 39), change in HIT-6 (9, 40), change in BDI-II (10, 41), and change in BAI (47). These outcome measures will be repeatedly assessed at weeks 0, 4, and 16 by a researcher who is blinded to participant allocation. Additionally, the BDI-II and BAI will be repeatedly assessed at weeks 8 and 12. Neuropsychological and others assessment scales will be conducted by the researcher in a quiet room. To guarantee consistency, the same researcher will perform all the outcome assessments.

#### Headache diaries

A headache diary was verified in a previous study (48). In the headache diary, the participants will document each headache onset in detail, including attack frequency, migraine duration, migraine days, pain characteristics, pain intensity, location, inductions of headaches, concomitant symptoms (nausea, vomiting, photophobia, and phonophobia), aggravation by/causing avoidance of routine physical activity (walking or climbing stairs), precipitating factors, and acute medications. Pain intensity will be evaluated using a VAS (0 to 10).

#### HIT-6

HIT-6 assesses six headache-related domains affected, including pain, social functioning, role functioning, vitality, cognitive functioning, and psychological stress, and is designed to offer a global evaluation of the negative impact of headaches (49, 50). The six questions are assigned a point value of 6, 8, 10, 11, or 13 based on five questionnaire items that indicate how frequently headaches disrupt with daily activities or induce stress (never, rarely, sometimes, very often, or always). The total score ranges from 36 to 78, with higher scores indicating higher levels of headache impact. A previous study confirmed that HIT-6 is a robust and accurate measure for differentiating headache impact between episodic and chronic migraines (51).

#### MSQ

The 14-item MSQ is a patient-reported outcome instrument that assesses the impact of migraine across three domains (52, 53): the role restrictive (MSQ-RR), the role preventive (MSQ-RP), and the emotional functioning (MSQ-EF). Items are assigned scores of one to six (none of the time, a little bit of the time, some of the time, a good bit of the time, most of the time, and all of the time). Raw domain scores are calculated as a sum of 14-item and rescaled from a 0 to 100-point scale, with higher scores indicating better quality of life. The MSQ is a reliable and accurate assessment that could distinguish between the functional effect of chronic and episodic migraine in the chronic migraine population (54).

#### BDI-II

The BDI-II is a 21-item self-report scale that evaluates the depression severity (55–57). Its contents is compatible with the diagnostic criteria for major depressive disorder (MDD) in the fourth edition of the Diagnostic and Statistical Manual of Mental Disorders (DSM-IV) (58). Items are graded on a four-point Likert scale ranging from zero to three, and the total score ranges from 0 to 63, with higher scores indicating higher levels of depression. The scale's features include its sufficient internal consistency, content reliability and worldwide dissemination (59).

#### BAI

The BAI is a 21 item self-report scale used to evaluate anxiety severity. This inventory was published in the USA by Beck in 1988 and is an anxiety assessment which has been carefully constructed to minimize misunderstanding with depression (60). The BAI examines affective, cognitive, and somatic components of anxiety. Items are rated from zero (not at all) to three (severely) depending on the severity of anxiety symptoms. The maximum total score is 63, with higher scores indicating greater anxiety. The BAI could be used as an effective assessment approach to determine between people who have and do not have panic disorder (61).

#### MRI outcome measures

This trial will concentrate on the following MRI indicators (week 0, week 4, and week 16): (1) fMRI: amplitude of low-frequency fluctuation (ALFF), regional homogeneity (ReHo), independent component analysis (ICA)-based functional connectivity (FC), seed-based FC, degree centrality (DC), (2) sMRI: voxel-based whole-brain voxel-mirrored homotopic connectivity (VMHC), cortical thickness, gray matter volume (GMV), white matter volume (WMV), total brain volume (TBV), (3) DTI: mean diffusivity (MD), radial diffusivity (RD), axial diffusivity (AD), and fractional anisotropy (FA).

### Safety assessments

Adverse events (AEs) are unintentional injuries or complications following intervention. At each visit, participants will be asked to disclose any aberrant responses or uncomfortable feelings experienced to the researchers. To evaluate the risks associated with acupuncture, any AEs will be tracked and documented in the CRFs. Needle-related AEs include needle sticking, bleeding, hematoma, subcutaneous hemorrhage, serious pain, dizziness, local infection, neurological symptoms, and fainting (62). All unexpected AEs will be recorded in detail in the CRFs, including the time of occurrence (transient or persistent), severity of AEs (mild, moderate, or serious), and possible causes (needle-related or non-needle-related) by participants and outcome assessors. All AEs will be systematically collected *via* semi-structured and open-ended questionnaires and categorized by acupuncturists and neurologists within 24 h (63). Different types of AEs occurring in one participant will be classified as independent AEs. One type of AE with multiple occurrences in each participant will be classified as one AE. Disagreements will be resolved through discussion and consensus.

Participants experiencing mild to moderate AEs will be treated immediately and constantly monitored by the attending acupuncturist. In the case of serious AEs, the research team will immediately provide medical advice to the participant within 48 h. Serious AEs shall be presented within 24 h to the principal investigator and the research ethics committee. AEs will be tracked until they are properly resolved. The research ethics committee will decide whether the trial should be terminated.

### Data management and monitoring

Clinical information from participants will be recorded using a CRF and headache diaries, which will be labeled with a specific number identification and documented by a researcher. A research assistant will check the clinical information for completeness, reliability, and consistency. Clinical information from the CRF and headache diaries will be entered into the EpiData database. Double data entry and validation will be performed by two investigators for reducing data input errors, and value pairs will be reviewed for discrepancies by referring to the paper-based source.

All CRFs and original paper-based data will be safely stored in locked filing cabinet. Electronic files will be saved in a password lock computer with restricted assessed to our study team members. Essential documents will be stored for a minimum of 5 years after the last publication. Additionally, the Center for Advancing Quality in Clinical Research of Beijing Hospital of Traditional Chinese Medicine will monitor the trial to assure enrollment rate, data accurateness and reliability.

### Sample size calculation

There is no acknowledged standard for estimating sample size in neuroimaging research. According to the demands of neuroimaging research (64, 65) and similar previous research (66, 67), a sample size of 12–26 persons in each group is an appropriate sample size for data processing. Desmond et al. found that a sample of 25 participants in each group are required to keep 80% power after multiple comparisons, and this power calculations were performed through the simulation-based method for group-level fMRI studies (68). In sum, a sample of 30 participants in each group is deemed acceptable to account for 10–20% participants dropout and incorrect images owing to head movements in this study.

### Retention strategies for participant engagement

The dropout is a critical determinant of results in acupuncture clinical trials, due to its time costing, more complicated procedure and even fear of needling (69). To avoid high dropout rate, we applied a flexible treatment timetable and personally illustration of acupuncture needle and effect from our senior investigator (70). However, participants will still be able to withdraw from this research at any time, and with or without any reason.

### Statistical analysis

#### Clinical data analysis

The analyses will be completed using Statistical Package for Social Science (SPSS) for Windows (version 22.0) by statisticians blinded to the group allocation and treatment procedure. The statisticians involved in the study are affiliated to the Research Center of Clinical Epidemiology, Peking University Third Hospital. All statistical analyses will be based on the intention-to-treat principle. The ITT principle in this trial will be comprised of all allocated participants who receive at least one treatment and have a complete baseline assessment. The last observation carried forward approach will be used to fill the missing data from participants who drop out. Next, the per-protocol principle will be used for sensitivity analyses, including all randomized participants who complete all the treatment and have MRI scans. The histograms, normal probability plots and a Shapiro-Wilk test will be conducted to evaluate the normality of the data. Standard deviations, and 95% confidence intervals will be calculated using the distributed data. All hypothesis tests will be two-tailed analyses with statistical significance defined as *p*-value <0.05.

The study will report the median (interquartile range) or mean ± standard deviation for continuous variables, and numbers (percentages) for categorical variables. The chi-square and Fisher exact tests will be performed to compare dichotomous variables. The independent two-sample *t*-test or Wilcoxon rank-sum test will be performed for normally or non-normally distributed continuous variables. A linear mixed-effects model will also evaluate multivariate-adjusted treatment effects across groups. Furthermore, we will take sex, age, and baseline migraine days as covariates and conduct a mixed-effects model to avoid the influence of these factors on the results.

#### MRI data analysis

An independent MRI analyst (LZha), who is not involved in the enrollment or assessment of participants, will perform the MRI data analysis. First, the fMRI data will be preprocessed and analyzed using FMRIB Software Library (FSL; http://fsl.fmrib.ox.ac.uk/fsl), Analysis of Functional NeuroImages (AFNI; http://afni.nimh.nih.gov/afni), and FreeSurfer (http://surfer.nmr.mgh.harvard.edu/). The sMRI data will then be analyzed using the voxel-based morphometry (VBM) toolbox within Statistical Parametric Mapping 12 (SPM12; http://www.fil.ion.ucl.ac.uk/spm), and the DTI data will be processed using FSL. Pearson's correlation between the changes in brain activity and improvement in clinical outcomes will be calculated in each group.

#### Brain connectivity analyses

T2^*^-weighted BOLD images will be preprocessed, including cardiorespiratory artifact correction (AFNI, RETRO-ICOR), head motion correction (FSL, MCFLIRT), and brain extraction (FSL, BET). Cortical surface reconstruction will be completed to improve structural-functional co-registration using boundary-based registration (FreeSurfer, bbregister). Functional data will then be registered to the MNI (Montreal Neurological Institute) space (FSL, FNIRT). Spatial smoothing and high-pass temporal filtering will also be applied. After the preprocessing procedure, different analytical methods will be used to examine the neural response, including ALFF, ReHo, seed-based FC, and ICA-based FC. These are the most common analysis indicators in fMRI that reflect the functional activity of the brain (71, 72).

#### VBM analysis

T1-weighted structural MRI data will be used in the SPM-based VBM analysis. Non-uniformity correction, tissue classification, and image registration will be incorporated in a generative model. The Diffeomorphic Anatomical Registration Through Exponentiated Lie Algebra (DARTEL) toolbox will be performed to generate normalized images, and then brain tissue will be segmented into gray matter, white matter (73). The normalized data will be smoothed and registered to the MNI space. Different analytical methods will be used in the trial, including DC, VMHC, cortical thickness, GMV, WMV, and TBV, to measure differences in the brain structure of participants (74).

#### DTI analysis

The Brain Extraction Tool (BET) in FSL will be conducted for brain extraction. Then, the eddy current distortion and head motion will be rectify using FMRIB's Diffusion Toolbox (FDT). FA, MD and L1, L2, and L3 eigenvalue maps will be calculated by fitting a tensor model using FDT. These FA images will be non-linearly registered to the FMRIB58-FA standard-space template and aligned to the MNI space. Different analytical methods will be used in the trial, including AD, FA, MD, and RD. These indices could be used to assess the cerebral structure's integrity and connection (75).

#### Predict treatment response from baseline MRI

Our study will analyze neuroimaging data utilizing a training-validation-testing approach with a nested cross-validation and feature selection routine to categorize MWoA patients into acupuncture responders and non-responders. The proportion of responders, defined as participants with a reduction in monthly headache onsets by at least 50% will be determined (33). We will concatenate all the brain features (including white matter, gray matter, cortical thickness, and resting FC) to produce a joint feature vector to feed into the subsequent analysis process. Machine learning approaches including linear support vector machine (LSVM), k-nearest neighbors (k-NN), and random forests (RF) will be used to select the features with the highest discriminative power for the classifier. To avoid the risk of overfitting, we will use a leave-one-out cross-validation strategy to evaluate the performance of the classifiers and feature ranking. The performance of each classifier will be estimated and compared by accuracy, sensitivity, and specificity. Sensitivity and specificity indicate the proportion of correctly classified responders and non-responders, respectively. Then the best classifier will be chosen.

### Patient and public involvement

The participants and the general public have no involvement in the study's design or implementation, and they will not be requested to provide feedback on reporting or to disseminate the finding. Participants will be able to view the study results *via* social media.

### Ethics and dissemination

The trial protocol adheres the principles of the Declaration of Helsinki and was approved by the Research Ethical Committee of Beijing Hospital of Traditional Chinese Medicine, Capital Medical University (Ethics Reference Number: 2020BL02-053-01) (Supplementary material). This trial was registered with the Chinese Clinical Trial Registry (ID: ChiCTR2100044251).

If any modifications or decisions are made, the ethics committee will review and approve amendments, and new protocols will be uploaded to www.chictr.org.cn. The authors will retain full control of the manuscript content. The study results will be published in a peer-reviewed academic journal and disseminated electronically *via* conference presentations.

## Discussion

### The possibility of acupuncture to modulate abnormal cerebral activity to treat MWoA

Brain imaging has recently been widely employed in acupuncture research as a promising approach for investigating the neural mechanism of acupuncture (76). In latest years, studies have focused more on the interactions between the FC networks of brain regions to determine the neural circuitry underlying the therapeutic benefits of acupuncture, rather than the alterations in a specific brain region during acupuncture (28).

However, the exact pathophysiology of migraine remains unclear, and there is an abundance of imaging research indicating extensive structural and functional changes in brain regions and networks. They might influence migraine pain experience and multisensory integration (77, 78). Therefore, utilizing these brain imaging approaches, rigorous design and implementation might facilitate research of acupuncture mechanisms in migraineurs.

After acupuncture therapy, research showed enhanced ReHo/ALFF/brain metabolites in brain regions involved in pain processing, such as the affective-emotional processing of pain [e.g., the insula (79, 80), cerebellum (79), and brainstem (79)], cognitive pain processing [e.g., the orbitofrontal cortex (80–82)], and the descending pain modulation system (DPMS) [e.g., the rostral ventromedial medulla/trigeminocervical complex (81)]. Meanwhile, research showed lower levels of ReHo/ALFF/brain metabolites in brain regions implicated in cognitive pain processing [e.g., the hippocampus (79, 80)] and in the resting-state network, such as the default mode network (DMN) [e.g., the posterior cingulate cortex (79, 80), precuneus (79), and inferior parietal lobule (79)], and frontal parietal network (FPN) [e.g., the postcentral gyrus (79, 80)].

After long-term acupuncture therapy, enhanced FC has been identified in the DMN (27, 83) and FPN [e.g., the superior frontal gyrus and medial frontal gyrus (83)]. Moreover, it has been reported that there is an enhanced FC between the right FPN (RFPN), the left FPN [e.g., the left precentral gyrus and left postcentral gyrus (84)] and DMN [e.g., the left inferior parietal lobule (84) and posterior cingulate cortex (85)]. After acupuncture treatment, a group of researchers (85) found a reduction in FC between the RFPN and DMN (right precuneus). Furthermore, they applied seed-based analysis (SBA) by using the right precuneus as a seed to investigate the involvement of the right precuneus during acupuncture. They revealed the enhanced right precuneus FC with the DMN, DPMS, RFPN, and the reward system. The right ventrolateral periaqueductal gray was selected as the seed in another research (84), the authors discovered greater FC of the ventrolateral periaqueductal gray with the limbic systems and the DPMS.

### Multimodal MRI as a promising approach to investigate the neural mechanism of acupuncture

A combination of two or more MRI scans is referred to as multimodal MRI. It has a high spatial-temporal resolution as well as broad anatomical and emotional sensitivity (86). Three of the most commonly employed neuroimaging modalities in acupuncture research are fMRI, sMRI, and DTI (87). Multimodal MRI may yield longitudinal approach to analyze the central mechanisms underpinning acupuncture's effects on MWoA. We aim to explore the neuropathological changes in patients with MWoA from the perspective of brain structure and function. sMRI and DTI may identify structural alterations in brain regions, while fMRI may investigate alterations in functional connectivity between brain regions.

The application of multimodal MRI and multidimensional analytic methodologies will be used to elucidate on the neural mechanisms of acupuncture from distinct brain regions and functional networks. Finally, cross-validation of fMRI, sMRI, and DTI data strengthens the results' accuracy and reproducibility.

### Neuroimaging predictor of acupuncture outcome in the treatment of MWoA

Treatment personalization is an important trend in medicine. Imaging data is widely being used to support in disease diagnosis for oncology (88) and psychology (36–38). Nevertheless, limited studies have applied imaging to predict acupuncture effect. Our previous research employed a machine learning classification approach to develop a prediction model of acupuncture effectiveness in migraineurs based on pretreatment gray matter (GM) structure. The model was 83% accurate in differentiating between acupuncture responders and non-responders (35). Our findings present an objective biomarker for acupuncture therapy response, as well as a novel strategy for developing personalized medication for migraine.

Differences between individuals in the effectiveness of acupuncture are a prevalent issue. A recent study showed that only approximately 50% of patients had considerable relief of symptoms after 1 month of acupuncture therapy (35). As a result, predicting acupuncture response might lower medical expenses for those classified as non-responders. ADDIN EN.CITE. This study will use multiple non-parametric-regression-based machines learning approaches to discriminate between responders and non-responders. These packages of techniques are self-acting in learning patterns from data and no assumptions are demanded of the structure of the data. These techniques capture non-linear relationships in the data, and the interrelationship between predictors. Machine learning approaches are increasingly used for class prediction issues, which can help to avoid the pitfalls flaw of parametric regression models (ignoring non-linear influences of independent variables on the outcome, or ignoring interactions between independent variables) (89). LSVM, k-NN, and RF are commonly used approaches in machine learning, many researches have verified their promising performance for diseases prediction (90–93). We compare various methods to obtain a classification model with better accuracy, sensitivity, and specificity.

The present study has several limitations. First, sham acupuncture, designed as a minimally invasive needle on non-acupoints in our study, might have produced certain physiological effects. Minimally invasive needles are required to minimize the non-specific effects of sham acupuncture. However, in several previous studies, sham acupuncture produced non-specific effects in the control group. In the future, we will use a placebo needle in the sham group. However, patients may identify the placebo needle because real acupuncture is widely used in China. Moreover, placebo needles might cause a multitude of peripheral, segmental, and cerebral reactions to an unexpected extent (94–96). Our further studies should compare acupuncture with standard therapies rather than comparing it only to sham acupuncture. Second, this present study only included a single center; therefore, the reproducibility of this study cannot be confirmed. Subsequently, more centers will need to evaluate the reproducibility and robustness of our predictive model. Third, a standard prescription will be used to assess the effectiveness of acupuncture in this study. To minimize performance bias, this study will not employ personalized prescription based on the acupuncturist's experience (46). Fourth, we will not apply magnetic resonance spectroscopy (MRS) to investigate brain metabolites due to budget constraints (97). Therefore, we plan to add MRS to our investigations in the future.

To summarize, our study aims to evaluate the efficacy of acupuncture for MWoA. We hope to present solid evidence for the mechanisms of acupuncture therapy on MWoA using multimodal MRI data on brain structure and function. Moreover, machine learning technology will be used to predict the acupuncture responders for MWoA.

## Ethics statement

The studies involving human participants were reviewed and approved by Research Ethical Committee of Beijing Hospital of Traditional Chinese Medicine, Capital Medical University. The patients/participants provided their written informed consent to participate in this study. Written informed consent was obtained from the individual(s) for the publication of any potentially identifiable images or data included in this article.

## Author contributions

LL is a principal investigator of the study and is responsible for making final decisions on the trial design and manuscript preparation and conducted the study. JH and LL drafted the protocol. LL and YK participated in the design of the study and contributed to the revising the protocol manuscript. LZe was responsible for the statistical design of the study. YZ was responsible for MRI data acquisition. LZha and ZT provided MRI advice and made critical revisions. JS, YC, QC, YL, LZhu, and BL participated in data collection and were in charge of recruitment. LL is a principal investigator of the study and is responsible for making final decisions on the trial design and manuscript preparation. All authors contributed to the article and approved the submitted version.

## Funding

This work will be supported by the following funding sources: the Capital Health Development Scientific Research Project Excellent Young Talents (Capital Development 2020-4-2236), the Beijing Municipal Education Commission Science and Technology Plan General Project (KM202110025005), the China National Natural Science Foundation (82074179), the China Association for Science and Technology Young Talent Lifting Project (2019-2021ZGZJXH-QNRC001), the National Key Research and Development Plan (2019YFC1709703), and the National Administration of Traditional Chinese Medicine: 2019 Project of Building Evidence-Practice Capacity for TCM (No. 2019XZZX-ZJ002).

## Conflict of interest

The authors declare that the research was conducted in the absence of any commercial or financial relationships that could be construed as a potential conflict of interest.

## Publisher's note

All claims expressed in this article are solely those of the authors and do not necessarily represent those of their affiliated organizations, or those of the publisher, the editors and the reviewers. Any product that may be evaluated in this article, or claim that may be made by its manufacturer, is not guaranteed or endorsed by the publisher.

## Abbreviations

MWoA: migraine without aura
MRI: magnetic resonance imaging
fMRI: functional magnetic resonance imaging
sMRI: structural magnetic resonance imaging
DTI: diffusion tensor imaging
MSQ: Migraine-Specific Quality of Life Questionnaire
HIT-6: Headache Impact Test
BDI-II: Beck Depression Inventory
BAI: Beck Anxiety Inventory
NSAIDs: non-steroidal anti-inflammatory drugs
EAN: European Academy of Neurology
AAN: American Academy of Neurology
the UK: the United Kingdom
the USA: the United States of America
SPIRIT, Standard Protocol Items: Recommendations for Intervention Trials
CONSORT: the Consolidated Standards of Reporting Trials
STRICTA: Standards for Reporting Interventions in Clinical Trials of Acupuncture principles
HCs: healthy controls
ICHD-III, The International Classification of Headache Disorders: 3rd edition
SAS: Statistical Analysis System
RA: real acupoints
WHO: World Health Organization
SA: sham acupoints
CRFs: Case Report Forms
TR: repeat time
TE: echo time
FOV: field of view
BOLD: blood oxygenation level-dependent
ALFF: amplitude of low-frequency fluctuation
ReHo: regional homogeneity
ICA: independent component analyses
FC: functional connectivity
DC: degree centrality
VMHC: voxel-mirrored homotopic connectivity
GMV: gray matter volume
WMV: white matter volume
TBV: total brain volume
MD: mean diffusivity
RD: radial diffusivity
AD: axial diffusivity
FA: fractional anisotropy
MMDs: monthly headache days
VAS: Visual Analog Scale
RFR: the role function-restrictive domain
RFP: the role function-preventive domain
EF: the emotional functioning domain
MDD: major depressive disorder
DSM-IV: the fourth edition of Diagnostic and Statistical Manual of Mental Disorders
AEs: adverse events
SPSS: Statistical Package for Social Science
ITT: intention-to-treat
LOCF: last observation carried forward method
PP: per-protocol
SDs: Standard deviations
CIs: confidence intervals
SPM: Statistical Parametric Mapping
DPARSF: Data Processing Assistant for Resting-State fMRI
VBM: voxel-based morphometry
FSL: fMRI of the brain Software Library
AFNI: Analysis of Functional NeuroImages
DARTEL: the Diffeomorphic Anatomical Registration using Exponentiated Lie algebra
MNI: Montreal Neurological Institute
FDT: FSL's Diffusion Toolbox
LSVM: linear support vector machine
k-NN: k-nearest neighbors
RF: random forests
DPMS: descending pain modulation system
DMN: default mode network
FPN: frontal parietal network
RFPN: right FPN
SBA: seed-based analysis
GM: gray matter
MRS: magnetic resonance spectroscopy.
