# Supplementary material for: Neurological mechanism and treatment effects prediction of acupuncture on migraine without aura: Study protocol for a randomized controlled trial
Source: Front Neurol. 2022 Sep 8;13:981752. doi: 10.3389/fneur.2022.981752 (PMC9492888; doi:10.3389/fneur.2022.981752)
Supplement: Supplementary file 1 [file Data_Sheet_1.docx]

**Additional file 2**

**Supplementary Table 1.** Acupoint and description of acupoint location

| Location of real acupoints used in the real acupuncture treatment group | | |
| --- | --- | --- |
| **Real Acupoints** | **Location** | **Depth of insertion** |
| Baihui [GV20] | 5 cun directly above the midpoint of the posterior hairline, or at the midpoint of the line connecting the apexes of the two auricles. | Parallel insert 0.5-0.8 cun |
| Fengfu [GV16] | On the back of neck, 1 cun directly above the midpoint of the posterior hairline, directly below the external occipital protuberance. | Oblique insert 0.5–1.0 cun |
| Fengchi [GB20] | On the nape, below the occiput, at the level of Fengfu, in the depression between the upper portion of sternocleidomastoideus and trapezius muscle. | Oblique insert 0.8–1.2 cun |
| Taiyang [EN-HN5] | On the lateral border of the orbit, at the point of intersection of thecontinuations of the eyebrow and the lower eyelid in the lateral direction. | Perpendicular or oblique insert 0.3-0.5 cun |
| Hegu [LI4] | On the dorsum of the hand, radial to the midpoint of the second metacarpal bone. | Perpendicular insert 0.5-1.0 cun |
|  |  |  |
| Location of sham acupoints used in the sham acupuncture control group | | |
| **Sham acupoints** | **Location** | |
| Sham acupoint (1) | The medial side of the arm at the anterior border of the insertion of the deltoid muscle at the junction of the deltoid and biceps muscles. | |
| Sham acupoint (2) | Half way between the tip of the elbow and the axilla. | |
| Sham acupoint (3) | On the ulnar side of the arm, half way between the epicondylus medialis of the humerus and the ulnar side of the wrist. | |
| Sham acupoint (4) | The edge of the tibia (1–2 cm lateral and horizontal to the Zusanli [ST36]*). | |

*Zusanli [ST36], 3 cun directly below Dubi and 1 fingerbreadth lateral to the anterior border of the tibia; Dubi location, when the knee is flexed, the point is at the knee, below the patella, in the depression from the patella ligament.
